# Toll-like receptor 4-mediated endoplasmic reticulum stress induces intestinal paneth cell damage in mice following CLP-induced sepsis

**DOI:** 10.1038/s41598-022-19614-6

**Published:** 2022-09-10

**Authors:** Yijie Wang, Dapeng Zhang, Congxin Li, Xue Wu, Chen He, Xiaolin Zhu, Haiyan Zhao, Lingjie Mu

**Affiliations:** 1 Department of Intensive Care Unit, The First Affiliated Hospital of Kunming Medical University, Kunming, China; 2 Department of Internal Medicine, Kunming Meizhao Physical Examination Center, Kunming, China

**Keywords:** Infectious diseases, Intestinal diseases

## Abstract

A marked elevation of TLR4 was observed in various organs of septic mice. The mechanism of TLR4 in intestinal epithelial cell damage in sepsis remains unclear. CLP mice models were used to assess the role of TLR4 in intestinal Paneth cell damage by histological, polymerase chain reaction, western-blot analyses. The ileal expression of TLR4 was increased by more than five-fold after CLP. CLP significantly increased 7-day mortality and was associated with a higher murine sepsis score (MSS), closely related with increased TLR4 expression. Histological staining revealed that a reduced number of Paneth cells, accompanied by reduced lysozyme and defensin alpha 5(DEF-5) expression as detected by PCR. Of note, the expression levels of ATF6, XBP1 and CHOP increased in the ileal of the sepsis group. Meanwhile, the uncleaved p90 ATF6 was markedly reduced and cleaved p50 ATF6 was increased in the sepsis group. Intriguingly, The TAK-242 had improved intestinal mucosal injury, reduced the expression of ATF6, XBP1 and CHOP and relieved the cleavage of ATF6. We found that increased the expression level of TLR4 in the ileal of CLP mice promoted the depletion of Paneth cell and reduced LYZ and DEF-5 expression. Furthermore, our findings suggested that TLR4-mediated the hyperactivation of ER stress, via activating the ATF6/CHOP pathway, might be one of the mechanisms associated with Paneth cells loss and dysfunction during intestinal barrier impairment of sepsis.

## Introduction

Sepsis was characterized by a dysregulated immune response to infection leading to life-threatening multiple organ dysfunction syndrome (MODS)^1^, which still was a common critical disease. The global incidence of sepsis was significantly higher from 10 years ago, reaching 437 per 100,000 person-years, and the number of hospitalizations increased to 31.5 million person-years, even surpassing patients with acute coronary syndrome or stroke^2^. The mortality rate of sepsis was even much higher than coronary heart disease. Meanwhile, the mortality and treatment costs of sepsis were significantly associated with economic level, with mortality significantly lower in developed countries than in developing countries (17–30% vs. 30.9–87.9%)^3^. There were about 1.75 million sepsis patients in the United States per year, about 55% of whom went to ICU and cost nearly 60 billion dollars a year^4^. In 2021, a study from Sichuan province reported that the current situation of sepsis treatment in western China. The mortality rate of sepsis patients was as high as 57.5%^5^, and the per capita hospitalization cost reached 11,390 dollars. Thus, sepsis was a global public health problem, killing more than 5 million people worldwide every year, which was a major economic burden for patients and society.

Sepsis could easily cause multiple organ dysfunction (MODS), in turn, MODS tended to aggravate sepsis^6^. This was why sepsis was difficult to treat and had a high mortality rate. A previous large-scale study in the UK found that the mortality rate of single organ dysfunction in sepsis patients was 18.5%, however, if five organs dysfunction occurred, the mortality rate would reach 69.9%^7^. In clinical work, we found that organs containing epithelial barriers, such as gastrointestinal, pulmonary, kidney, liver were vulnerable to be affected by sepsis. The intestinal tract was the earliest and most vulnerable organ in sepsis, considered to be the "engine" of MODS of sepsis patients. The popular clinical saying "Win the gastrointestinal, win the world" was enough to see the important role of gastrointestinal function in the treatment of severe patients. The core of intestinal function was concentrated in the intestinal epithelial cells, which was one of the fastest updated and most dynamic tissues in mammals, and was considered to be the first line of defense against disease. Intestinal stem cells with leucine-rich repeat-containing G protein-coupled receptor 5 (LGR5 +) located in the crypt continuously differentiated into various cells, constantly renew the epithelial cell layer^8^. In the development of sepsis, intestinal epithelial cell apoptosis increased, while the migration of intestinal stem cells slowed and the differentiation and proliferation decreased, which increased the intestinal permeability^9^ and impaired the immune barrier function. Finally, various pathogenic factors leaved the gut through the portal vein blood flow and mesenteric lymph fluid, causing distal compartment organ damage and leading to MODS^10^. At present, many studies had clarified the complex and close links between the various types of cells in the gut and between cells and intestinal flora, but there were few studies on the specific functional damage mechanism of intestinal epithelial cells in sepsis, especially in intestinal stem cells.

Toll-like receptors (TLRs), a type I transmembrane protein belonging to the pattern recognition receptors 9 family, were a key regulator of natural and adaptive immunity and are highly expressed in sepsis. Recently, Krivan et al. reported an increased expression of TLR 2,3,4, and 7 mRNA in the kidney and intestine of sepsis mice^11^, which suggested that TLRs played an important role in the mechanism of concurrent organ dysfunction in sepsis. As a lipopolysaccharide receptor, TLR4 could induced prostaglandin E2 increase by cyclooxygenase 2, resulting in the proliferation of LGR5 + stem cells in the dividing intestinal crypts of neonatal mice^12^. At the same time, TLR4 could be expressed in Lgr5 + stem cells and promoted apoptotic signals to the mitochondria, thereby regulating cell proliferation and apoptosis by activating PUMA and interacting with antiapoptotic factors. While TLR4 also played an important role in regulating normal intestinal epithelial differentiation, reducing goblet cells in the intestine by activating notch signaling, which was unaffected by the gut microbiota. By inhibiting the TLR4-mediated NF-κB pathway, goblet cells in the intestinal villi could be protected to mitigate intestinal damage^13^. Notably, TLR4 could also be activated by certain stimulants, such as vitamin D, inducing the production of antimicrobial peptides in Paneth cells and enhancing the function of the intestinal epithelial barrier^14^. The role of TLR4 in different diseases or in different cells of the same disease was different, and the mechanisms were also very complex. Deletion of TLR4 accelerated the migration rate of intestinal stem cells in mice with necrotizing enterocolitis. However, in septic mice, TLR4 deficiency slowed down the migration rate of intestinal stem cells. For instance, TLR4 played a role in myeloid cells of septic mice by phagocytosis and efficient bacterial clearance, reducing inflammatory response and liver damage, and thus improving survival. In hepatocytes, TLR4 could effectively eliminate LPS from the circulation, but it would weaken the phagocytic capacity of macrophages, increase the bacterial levels and reducing the survival rate^15^. As early as 2006, Li M et al. found a novel cyclohexene derivative, ethyl (6R)-6-[N-(2-Chloro-4-fluorophenyl)sulfamoyl]cyclohex-1-ene-1-carboxylate (TAK-242), selectively inhibits TLR4-mediated cytokine production through suppression of intracellular signaling^16^. Thereafter, as a TLR4 signaling inhibitor, TAK-242 had been widely used in the related studies of TLR4-mediated inflammatory response. For example, pretreatment with TAK-242 could reduced or reversed TLR4-inducated endotoxemia-induced muscle wasting^17^. TAK-242 modulates the structure of the gut microbiota in colitis by downregulating TLR4 and increasing JAK2/STAT3 phosphorylation, which may be a novel therapeutic candidate for ulcerative colitis^18^. Therefore, this study started with TLR4 to explore the molecular and biological mechanism of intestinal Paneth cell injury in septic mice, and to provide a new theoretical basis and therapeutic targets for the prevention and treatment of intestinal function injury.

## Results

### Upregulation of TLR4 reduced survival in mice following CLP-induced sepsis

**Figure 1:**
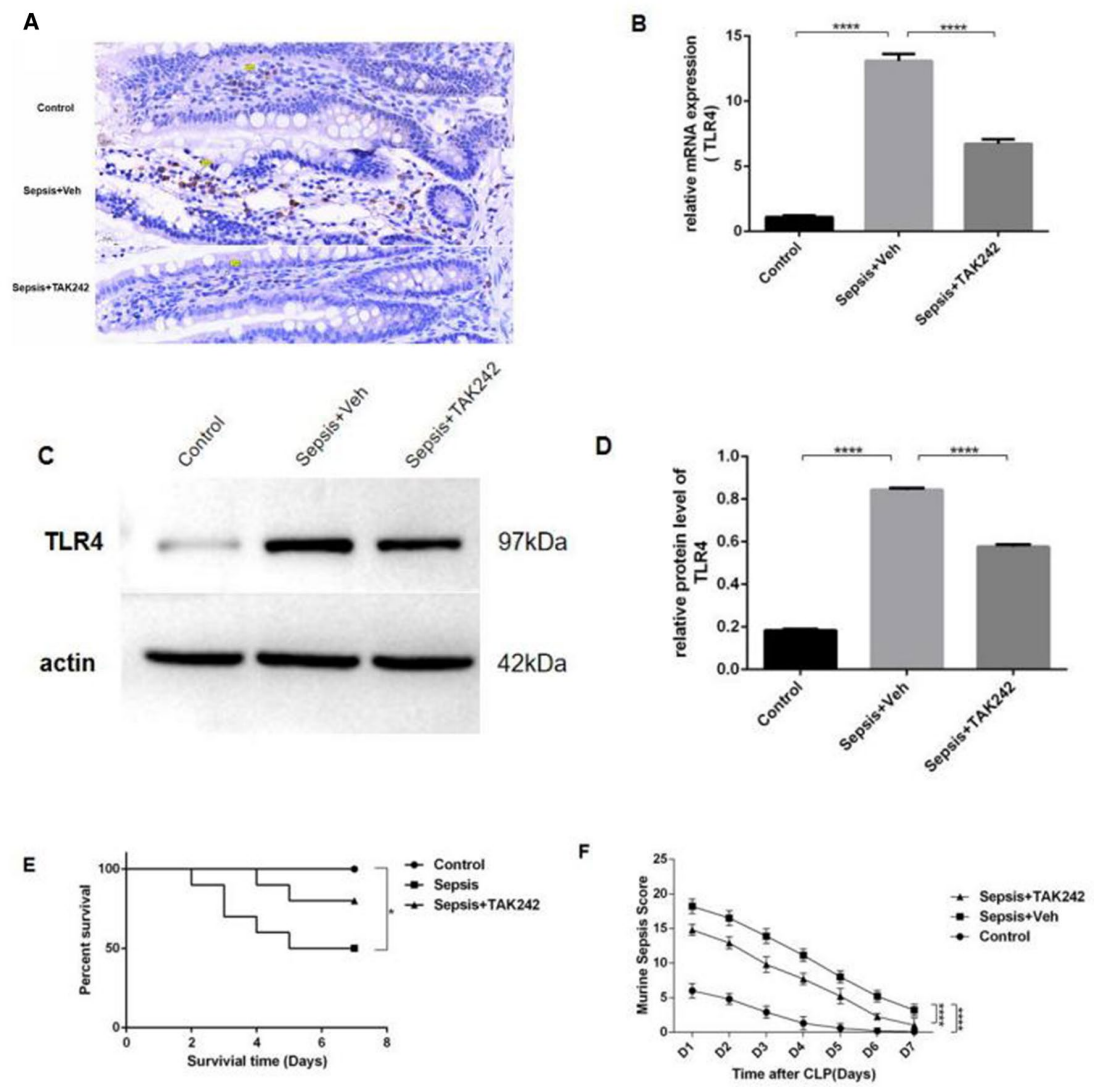
TLR4 was upregulated after cecal-ligation and puncture (CLP) induced sepsis in mouse from which small intestine, as demonstrated by (**A**) immunohistochemical, (**B**) real-time PCR and (**C, D**) westrenblotting for TLR4. CLP induced sepsis led to (**F**) reduced survival and (**E**) higher sepsis severity score (n = 10 mice). Error bars denote standard error of the mean. *P < 0.05; **P < 0.01; ***P < 0.001; ****P < 0.0001.

In the control group, all mice survived within 7 days, and the survival rate was 100%. However, in the sepsis group, five mice died within 7 days, and the survival rate was only 50% (*P* = 0.0212). At the same time, upregulation of TLR4 was significantly found in ileal tissue of the sepsis group (*P* < 0.0001) (Fig. 1A,B,C,D). Compared with the sepsis group, mice treated with TAK-242 had decreased 7-day mortality but no statistical differences (*P* = 0.346) (Fig. 1E), significantly lower Murine Sepsis Score (MSS) (*P* < 0.0001). (Fig. 1F).

### Upregulation of TLR4 caused the damage of intestinal mucosal structure in mice following CLP-induced sepsis

**Figure 2:**
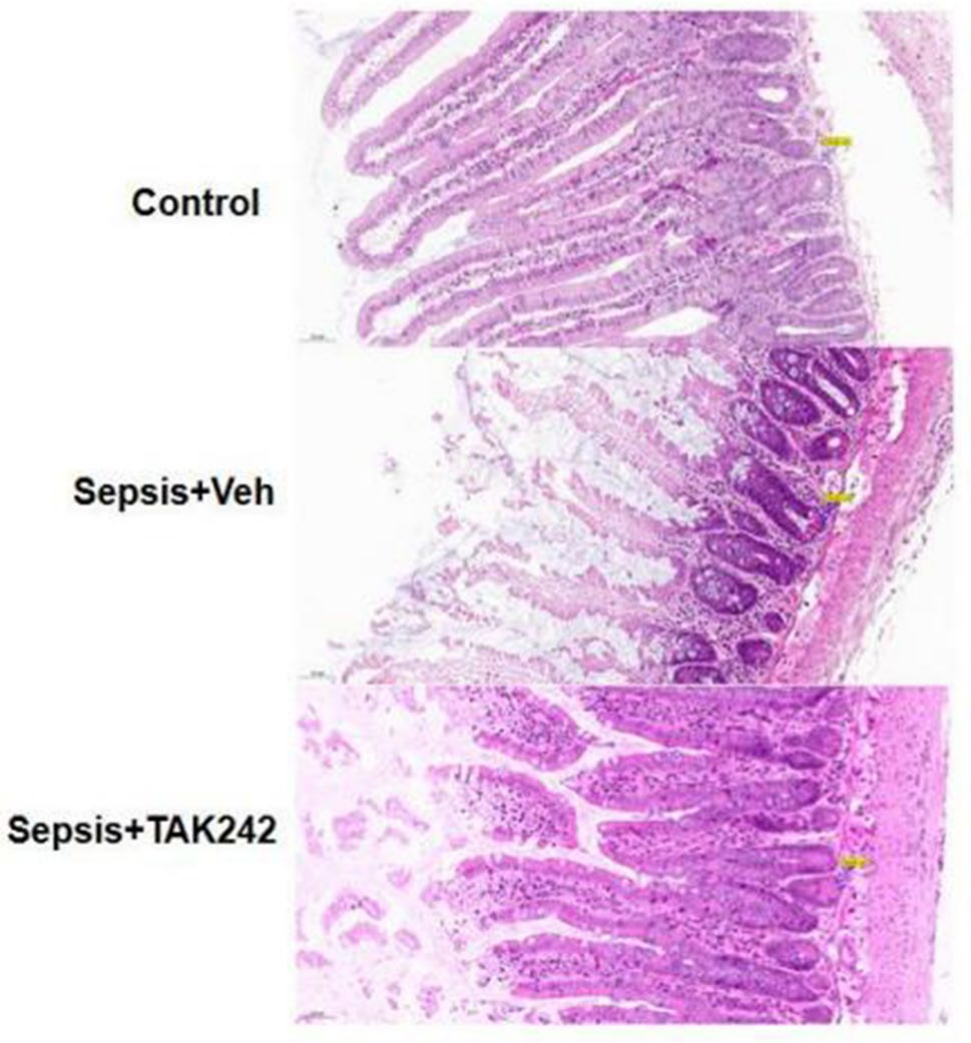
TLR4 caused the damages of intestinal mucosal structure in CLP mouse by Hematoxylin–Eosin staining, including intestinal epithelium degeneration and shedding, villus atrophy and disordered arrangement. Compared to the Sepsis group, TAK-242 treatment had reduced the damages, with a smaller necrotic shedding tissue, small intestinal villi thickening.

Histopathological changes were particularly visible in the ileal tissues stained by hematoxylin and eosin in the sepsis group, and the observed defects included intestinal epithelium degeneration and shedding, seriously damaged intestinal mucosa, villus atrophy and disordered arrangement, inflammatory cell infiltration in the mucosal layer, submucosal blood vessels dilation. Compared to the sepsis group, the intestinal mucosa structure was relatively complete, with a smaller number of inflammatory cells and necrotic shedding tissue, small intestinal villi thickening in the intervention group. However, compared with the control group, the intestinal mucosa in the treatment group still had necrosis and shedding, especially in the apical area of the small intestinal villus, villus disordered arrangement (Fig. 2).

### Upregulation of TLR4 resulted in Paneth cell disruption

**Figure 3:**
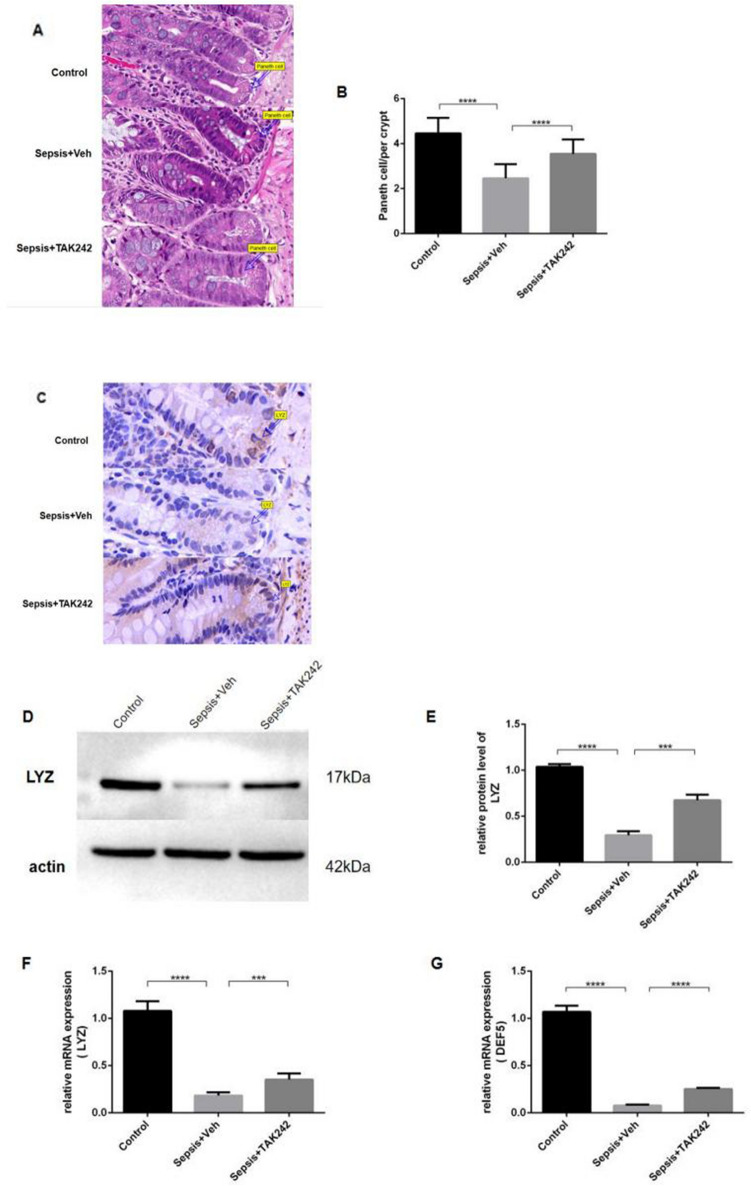
Reduced numbers and depressed secretory function were found in Paneth cells in CLP mouse. (**A**) Histological analysis of the alterations in Paneth cell number and morphology. Paneth cells were indicated by arrows. Magnification, × 20. (** B**) Quantification of the number of Paneth cells in the different groups, as assessed by hematoxylin and eosin staining. LYZ was performed to observe the function of Paneth cells. The difference in the expression of LYZ among groups were detected by (**C**) Immunohistochemical staining (brown, Magnification, × 20), (**D,E**) westernblotting and (**F**) PCR. (**G**) The expression of mRNA DEF-5 was compared in diffrent groups. *P < 0.05; **P < 0.01; ***P < 0.001; ****P < 0.0001.

Intestine epithelial cells were apoptotic and shed in sepsis. Paneth cells, as a key point, maintain gut mucosal integrity and homeostasis. In the present study, histological analysis was performed to assess the presence of Paneth cells. Paneth cells containing granules were observed at the base of the intestinal crypts in the control group. Mice in the sepsis group exhibited significantly fewer Paneth cells in the crypt (*P* < 0.0001), with disorganized granules or without granules. However, the numbers of Paneth cells and granule morphology were partly augmented in intervention group (*P* < 0.0001). (Fig. 3A,B) To further investigate the secretory function of Paneth cell, Immunohistochemical staining (Fig. 3C), western blot (Fig. 3D,E) and real-time quantitative PCR (Fig. 3F,G) were also performed targeting Lysozyme(LYZ) and α-defensins (HD-5), which were two kind of antimicrobial peptides (AMPs) secreted by Paneth cell. In the sepsis group, the expression levels of LYZ and HD-5 (both *P* < 0.001) were significantly reduced as compared to the control counterparts. However, among mice treated with TAK-242, the expression levels of LYZ (*P* = 0.0009) and HD-5 (*P* < 0.0001) had more than two-fold increased as compared to mice in the sepsis group.

### The damaging effect of TLR4 on Paneth cell was caused through ER stress

**Figure 4:**
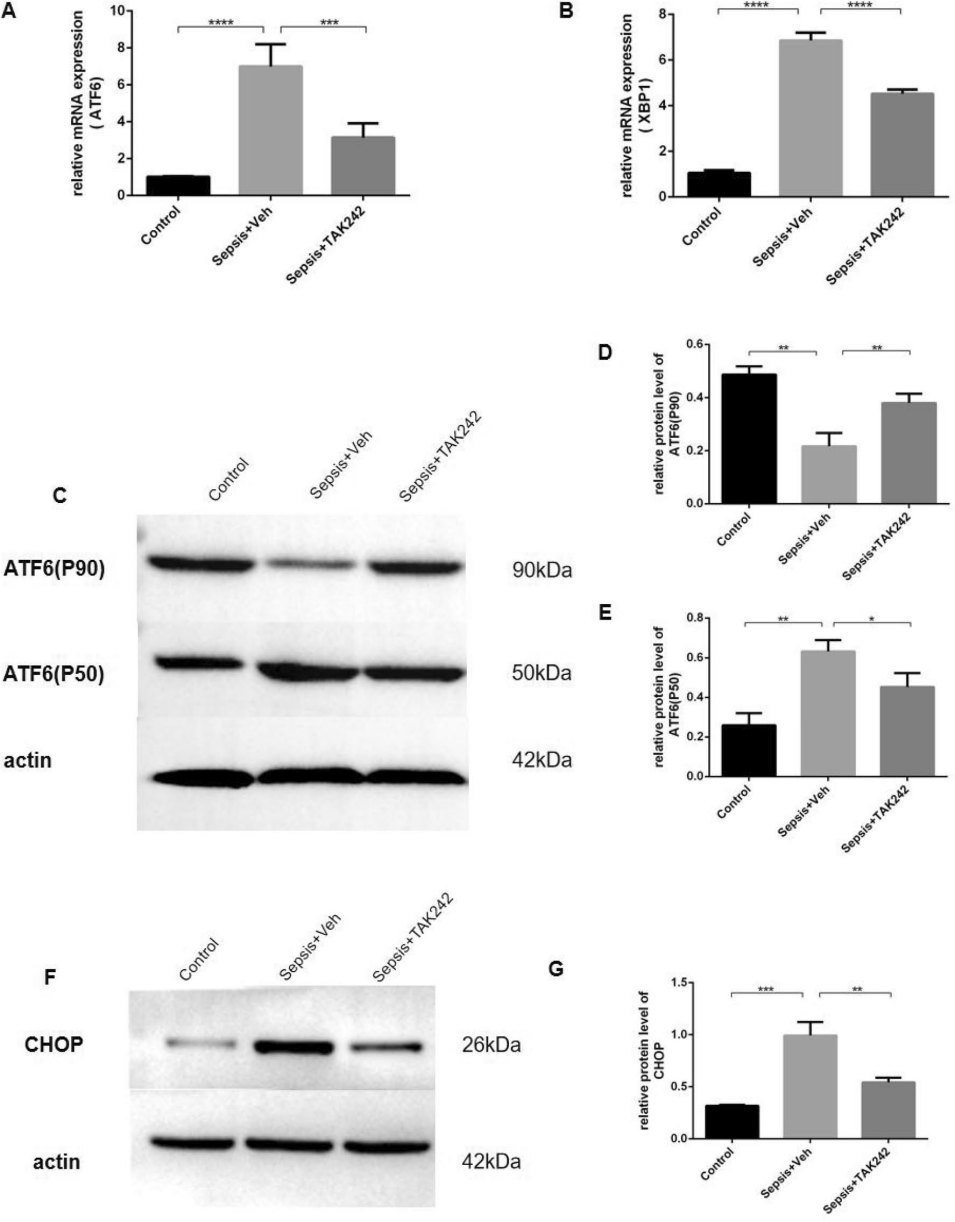
The ER stress was activated in the small intestine of CLP mouse. While TAK-242 treatment attenuated the activation of ER stress. (**A**,**B**) The expression of mRNA ATF6 and XBP1 were significantly increased in the sepsis group and dramatically decreased in the treatment group by PCR. Furthermore, sepsis of CLP induced the cleavage of ATF6 in the small intestine. The uncleaved (90 kDa) and cleaved (50 kDa) forms of ATF6 were detected by western blot assay. As illustrated in (**C**,**D**,**E**), the p90 ATF6 band was markedly reduced, with a concomitant increase in the p50 ATF6 component in the sepsis group. After treatment with TAK-242, the cleavage of ATF6 was significantly reduced compared with the sepsis group. (**F**,**G**) As an activated ER stress responsive gene, the protein levels of CHOP was markedly increased in the sepsis group and reduced in the treatment group by western blotting. *P < 0.05; **P < 0.01; ***P < 0.001; ****P < 0.0001.

To further investigate the underlying mechanisms of the damaging effect of TLR4 on Paneth cell, because of a complex relationship with TILR4 and Paneth cell, ER stress was adopted in the study by PCR and western blot targeting three sensor pathways. The present results suggested that compared with the control group, the result of real-time PCR revealed the mRNA expression level of ATF6 and XBP1 both were increased in the sepsis group (both *P* < 0.0001) and dramatically decreased in the treatment group (*P* = 0.0003, *P* < 0.0001, respectively) (Fig. 4A, B). Furthermore, CLP-induced sepsis induced the cleavage of ATF6 in the small intestine. The uncleaved (90 kDa) and cleaved (50 kDa) forms of ATF6 were detected by western blot assay. As illustrated in Fig. 4C,D,E, the protein expression level of p90 ATF6 was markedly reduced, with a concomitant increase in the p50 ATF6 component in the sepsis group (*P* = 0.0014, *P* = 0.0015, respectively). After treated with TAK-242, the cleavage of ATF6 was significantly reduced compared with the sepsis group (*P* = 0.0098, *P* = 0.0261, respectively). As an activated ER stress responsive gene, the protein level of CHOP was markedly increased in the sepsis group (*P* = 0.0009) and reduced in the treatment group (*P* = 0.0047) by western blotting (Fig. 4F,G).

## Discussion

Gut was hypothesized to be the motor of the progression of sepsis and multiple organ dysfunction syndrome, as a proinflammatory organ per se that drives the systemic inflammatory response associated with MODS. Striking, one of the most important pathways that mediates the balance between injury and repair in the intestine, and that plays a key role in the perpetuation and aggravation of sepsis pathogenesis, was TLR4, which recognized multiple pathogen-associated molecular patterns (PAMPs) from bacteria, viruses, and other pathogens^19^, specifically lipopolysaccharide (LPS) on gram-negative bacteria^20^. TLR4 was important not only in initiating inflammatory responses, its overstimulation can be detrimental leading to hyperinflammation, but also in the innate immune^21^. During early sepsis, cell-wall components from bacteria activated TLR4 resulting in a “cytokine storm” of pro-inflammatory mediators generated, mainly via the mitogen-activated protein kinase and NF-κB pathways^22^. Subsequently, alterations of the intercellular contacts (adherens junction and tight junction) caused intestinal hyperpermeability^23^. Meanwhile, TLR4 is significantly higher in crypts and lower on surface epithelium, which is positively correlated with the high expression of IL-6 in the lamina propria^24^. The overexpression of TLR4 prompted commensal bacteria exploit transcytotic pathways to cross the intestinal epithelium in a TLR4-dependent manner^25^. However, Non-infectious factors can also induced gut epithelial barrier dysfunction and subsequent bacteria translocation even sepsis of gut origin are mediated by TLR4 signaling^26^. In addition, TLR4 can mediate hyperglycemia, insulin resistance and proinflammatory molecule production, decreasing ApoM gene expression and plasma ApoM, damage gut-vascular barrier (GVB), thereby aggravate the intestinal function^27^. In this study, we demonstrated that upon induction of sepsis by CLP in mice, the expression of TLR4 was increased by over five-fold, as previously demonstrated by Krivan et al.^11^. Although previous studies had shown that low level of expression of TLR4 was observed in small intestine IECs and higher expression of these same TLRs in colon IECs^28^. We also found that TLR4 was mainly expressed in the villous stroma in the small intestine. At the same time, we observed a marked atrophy and disordered arrangement of villus, an observably seriously damaged intestinal mucosa accompanying by the degeneration and shedding of intestinal epithelium with increased TLR4 in the small intestine. The reason underlying this might be that sepsis induced upregulated of apoptosis in intestinal epithelial cells^29^, a profound decrease in crypt proliferation^30^ and mucus secretion. The aforementioned pathological changes in the small intestine were reversed after treatment with TLR4 receptor antagonists, which could reduce the activation of nuclear transcription factors, inhibit the secretion of inflammatory factors, protect tight junction protein, and protect the intestinal mucosal barrier through multiple pathways^31^. The results of this study similarly showed that TAK-242 treatment mice had better survival after CLP and a better MSS score, too.

As the guardian of small intestinal crypts, stem-cell derived Paneth cell not only feed into a delicate communication loop which aims at propagating stem cell function and an epithelial regenerative response^32^ but rather secrete antimicrobial peptides (AMPs), for example, α-defensin and lysozyme, which have a high antimicrobial activity, to link this specialized intestinal epithelial cell line to innate immune functions^33^ and control gut microbial communities^34^, finally confer intestinal protection from pathogenic insults^35^. As such, Paneth cell as a site of origin for intestinal inflammation play a crucial role in intestinal homeostasis and barrier function. In the present study, we investigated that Paneth cells were significantly ablated in the crypt in the sepsis group, with exhibited significantly fewer LYZ and HD-5, which was inversely correlated with TLR4 expression levels in small intestinal epithelial cells. In line with our study, Yu et al. reported that sepsis inhibited the function of Paneth cells and goblet cells with reduced secretion of lysozyme and suppressed autophagy, thus potentially contributing to barrier dysfunction in intestinal pathologies^36^. As we all known, TLRs had been shown to regulate epithelium permeability or secretion of defensin by Paneth cells, which acted as sensors of pathogen associated molecular patterns were required for Paneth cell degranulation. The TLR4 agonists LPS induced late degranulation mediated by TNF-α^37^. Similarly, previous studies suggested that only TLR4 was detected in the secretory granules of Paneth cells. A reduction of TLR4-positive secretory granules and the formation of TLR4-positive vacuoles were found in the ileal Paneth cells under the hyper-proliferation of indigenous bacteria^38^. But in our experiments, we found that the change in the expression level of TLR4 inversely correlated with the number and function of Paneth cells, and TLR4 inhibitors (TAK-242) could increase Paneth cell numbers, improve the function of Paneth cells secreting lysozymes and HD-5, and protect the intestinal barrier. We proposed that excessive TLR4 activation might lead to impair the function of Paneth cells.

To find out the further reasons, in the present study, the expression levels of protein associated with endoplasmic reticulum (ER) stress were investigated with the aim to determine whether the ER stress response inducing by TLR4 signaling is associated with Paneth cells loss or dysfunction in sepsis. The ER, which is the organelle responsible for protein folding and assembly, is activated a self-protective response by the accumulation of misfolded or unfolded proteins, described as the ER stress response in order to maintain cellular homeostasis. TLR4 signaling induces ER stress in intestinal stem cells, which leads to their apoptosis and initiates the development of NEC^39^. As the major secretory cells in the gut, Paneth cells exploit ER stress response to sufficiently express and secrete different types of antimicrobial peptides(AMPs) through secretory autophagy^40^. Nevertheless, prolonged ER stress triggered in Paneth cells may lead to persistent apoptosis of intestinal epithelial cells, causing the disruption of the mucosal barrier function and proinflammatory responses. Liu et al. reported that the increased ER stress was associated with Paneth cells loss. At the same time, Paneth cell depletion further aggravated intestinal ER stress and the intestinal mucosal damage during acute necrotic pancreatitis (ANP)^41^. Targeted inhibition of ER stress by 4-phenylbutyric acid may inhibit inflammatory responses and Paneth cells loss, alleviating the symptoms of ANP^42^. The ER stress sensor pathways, including IRE1/sXBP1, PERK/EIf2α and ATF6, orchestrate the major regulatory circuits to ensure ER homeostasis^43^. Normally, the chaperone BiP kept the three sensors in their inactive status. When misfolded proteins accumulated within the ER lumen, BiP dissociated from PERK, IRE1 and ATF6, which led to their activation. We observed that compared with the control group, the mRNA levels of ATF6 and XBP1 were elevated in the sepsis group. Furthermore, the uncleaved (90 kDa) and cleaved (50 kDa) forms of ATF6 were detected by western blot assay. We found that sepsis of CLP induced the cleavage of ATF6 in the small intestine. The cleaved soluble transcription factor p50 ATF6 was significantly increased in the sepsis group, then free to translocated to the nucleus where it subsequently activated ER stress responsive genes, such as GRP78, GRP94, calreticulin, and CHOP^44^. We also observed that the protein expression level of CHOP was significantly upregulated in the sepsis mice. CHOP is a major pro-apoptotic transcription factor that mediates ER-stress induced apoptosis^45^. Hetz, et al. reported that ATF6 was cleaved to become an active form and enter the nucleus to promote the transcription of ER-stress responder genes^46^. While another signaling pathway, IRE1 mediated the unfolded protein response (UPR) in part by regulating XBP1 mRNA splicing in response to ER stress. Sovolyova, et al. found that PERK/EIf2α induces cell death through the transcription factor CHOP in response to prolonged ER stress, which was similarly activated in the small intestine of sepsis mice^47^. More importantly, in this study we discovered TLR4 inhibitors(TAK-242) could improve various indicators of excessive ER stress, inculding downregulating the level of ATF6 mRNA, reducing the cleavage of ATF6 and in turn reducing the expression of CHOP compared with the sepsis group. Taken together, the present results suggested that activated the TLR4/ATF6/CHOP pathway of ER stress might be a cause of Paneth cells loss or secretory dysfunction of CLP mice, thereby alleviating intestinal mucosal damage and repairing barrier function.

One limitation of the present study was that small number of samples might lead to some differences not reflected, another imitation was the some signaling pathways of ER stress may be activated by increased TLR4, one of which is the main cause or the intersection of each other, requiring confirmation by additional studies^48^. Additionally, the present study was only performed to preliminarily examine the roles of TLR4 in Paneth cells in sepsis, but which might not be direct, and perhaps TLR4 mediated an interesting link between Paneth cells and intestinal stem cells. We would prepare to conduct cell experiments to further validate the positive results obtained in animal models. Furthermore, the therapeutic potential of strategies aiming to restrain the function of TLR4 to repair the intestine function of sepsis requires further validation.

## Conclusion

We found that increased the expression levels of TLR4 in the ileal tissues of sepsis mice exacerbated intestinal damage, increased mortality rates, promoted the depletion of Paneth cell and reduced the expression of LYZ and DEF-5. Furthermore, our findings suggested that TLR4-mediated the hyperactivation of ER stress, via activating the ATF6/CHOP pathway, might be one of the mechanisms associated with Paneth cells loss and dysfunction during intestinal barrier impairment of sepsis.

## Materials and methods

### Animals

Thirty male Wister mice, aged 7–8 weeks and weighing 250 ± 10 g supplied from the Kunming Medical University Experimental Animal Center. All the experimental protocols were approved by The Animal Care and Use Committee of Kunming Medical University and the experiments were performed in accordance with the institutional animal care guidelines. More specifically, animals were kept in separate cages under stable conditions (temperature 25 °C ± 1 °C, relative humidity 55% ± 5%, artificial day-night cycle of 12:12 h). All animals had free access to food and water was maintained prior to the experiments.

### Establishment of experimental septic mouse model

A clinically relevant mice model of sepsis was created by cecal ligation and puncture (CLP)^49^. At first, intraperitoneal anesthesia (using 10% Caldehyde at a dose of 300 g/ml), the abdominal area was shaved and disinfected by applying an antiseptic solution. A 1 cm midline abdominal incision was made and the cecum was exposed. The distal half of the cecum was later ligated with a silk suture and was twice punctured through with a 22-gauge needle allowing the release of fecal material into the peritoneal cavity. Finally, the cecum was placed back into the peritoneal cavity and the incision was closed in two layers with a 4.0 Vicryl suture. The severity of sepsis following CLP procedure was assessed based on murine sepsis severity (MSS) score^50^.

### Experimental design

Thirty mice were randomly divided into 3 groups as follows: (1) Control group (Control); (2) sepsis group (Sepsis + Veh); (3) treatment group (Sepsis + TAK-242), each consisting of 10 mice. The control group mice underwent a sham operation receiving a laparotomy without cecal ligation and puncture. The sepsis group and the treatment group mice underwent CLP operation. All animals were accessed to water and chow with free, in which the treatment group mice administered TLR4 inhibitor TAK-242 (3 mg/kg) via intraperitoneal injections^51^, and the control and the sepsis group mice administered equal volume drug solvent (0.9% sterile saline) through intraperitoneal injections, once a day for 7 consecutive days. At the end of the experimental period for each group, euthanasia was performed. Part of ileal tissue obtained were fixed in 4% paraformaldehyde for 24–48 h and paraffin embedded for histological analysis. The remaining portions were rinsed with distilled (deep treatment) water, flash-frozen with liquid nitrogen and stored to − 80 °C for mRNA and protein analysis.

### Histological and immunohistochemical analysis

The ileal tissues paraffin embedded were cut into 5 µm-thick sections placed on glass slides. Subsequently, those sections were stained by hematoxylin and eosin for 8 min at room temperature for the study and evaluation of tissue injury. In addition, the sections were cut in serial sections and were immunohistochemical stained using the following primary antibodies: anti-TLR4 antibody (1:200; Rabbit. no. PA5-23,124; ThermoFisher, Inc.) and anti-lysozyme antibody (1:250; Rabbit. no. PA5-114,441; ThermoFisher, Inc.). The number of Paneth cells in each crypt was determined using the ImageJ software (version 1.8.0; National Institutes of Health) analyzing eight fields per slide^41^.

### Real-time PCR

Total RNA was extracted from the ileal tissues by the Trizol lysate (15,596,026, Lifetech, USA) according to routine presequence treatment. For each specimen, RNA was reverse-transcribed into cDNA using FastKing RT Kit (With gDNase) FastKing cDNA (KR116 SYBR Green master mix: KAPA KK4601). Quantitative real time PCR was performed with LightCycler^®^480 Multiwell Plate 96, white using primers targeting TLR4, YZ, DEF-5, XBP1, ATF6 and β-actin. Relevant gene expression values were analyzed by the 2^−ΔΔCt^ method. All primers are synthesized by sangon (Invitrogen, Guangzhou, China). All the experiments were repeated 3 times under the same conditions.

The primer sequence is as follows:

TLR4 (R)-F: CAATCGCATAGAGACATC.

TLR4 (R)-R: GTTCAACATTCACCAAGA.

LYZ (R)-F: ATGTCTGGCTACTATGGA.

LYZ (R)-R: TTTCTGGCTTGTGTGTTA.

DEF-5 (R)-F: TCATGGAGGACCAGGATAT.

DEF-5 (R)-R: CCTTCACATCTGCATCTTG.

ATF6 (R)-F: TAATGGTGCTAAGTGAAGA.

ATF6 (R)-R: ACTCTGTCGTGTTAATGA.

XBP1 (R)-F: TGTTGCCTCTTCAGATTC.

XBP1 (R)-R: GAGTTCCTCCAGATTAGC.

β-actin (R)-F: GCAGGAGTACGATGAGTCCG.

β-actin (R)-R: ACGCAGCTCAGTAACAGTCC.

### Western blot

Ileal tissue samples were analyzed by western blot to determine the protein levels of TLR4, LYZ, ATF6 and CHOP. The samples were flushed with PBS and homogenized in RIPA lysis buffer that contained protease inhibitor. Protein concentration was determined by BCA assay (Biyuntian, China). Equal amounts of protein were loaded, and electrophoresis was applied on sodium dodecyl sulfate-polyacr-ylamide gel electrophoresis (SDS-PAGE). Proteins were transferred to PVDF membranes (Millipore), incubated overnight with anti-TLR4 (1:500, ThermoFisher PA5-23,124), anti-LYZ (1:1000, ThermoFisher PA5-114,441), anti-ATF6 (1:1000, Invitrogen PA5-20,215), anti-CHOP (1:1000, ThermoFisher MA5-32,571) and β-actin (1:2000, Zhongshanjinqiao TA-09) at 4 °C overnight. Then, the membranes were incubated with Horseradish peroxidase (HRP)-conjugated secondary antibody for 2 h at room temperature. Band intensities were semi quantitatively analyzed by ImageJ software, using β-actin bands as loading controls to normalize values.

### Statistical analysis

Statistical analysis was performed using the SPSS 21.0 software. All measures were represented using mean ± standard deviation (x ± s). Normality was assessed by using the Shapiro–Wilk (Royston) test. Mortality was compared by Kaplan–Meier survival curves and analyzed by the log rank test. Differences between groups were analyzed by t test and one-way analysis of variance (ANOVA) (alpha 0.05, two-tailed )followed by Bonferroni posttests (GraphPad Prism 6.0 Mac; GraphPad Software). Results were considered statistically significant when *p* < 0.05.

### Approval for animal experiments

All the experimental protocols were approved by The Animal Care and Use Committee of Yunnan Luoyu Biotechnology and the experiments were performed in accordance with the institutional animal care guidelines. The authors complied with the arrive guidelines.

## Supplementary Information

Supplementary Information 1.

Supplementary Information 2.

Supplementary Information 3.

Supplementary Information 4.

Supplementary Information 5.

Supplementary Information 6.

Supplementary Information 7.

Supplementary Information 8.

Supplementary Information 9.

## Data Availability

All data generated or analysed during this study are included in this published article (and its Supplementary Information files).
