# Supplementary figures and images for: Toll-like receptor 4-mediated endoplasmic reticulum stress induces intestinal paneth cell damage in mice following CLP-induced sepsis
Source: Sci Rep. 2022 Sep 10;12:15256. doi: 10.1038/s41598-022-19614-6 (PMC9464222; doi:10.1038/s41598-022-19614-6)

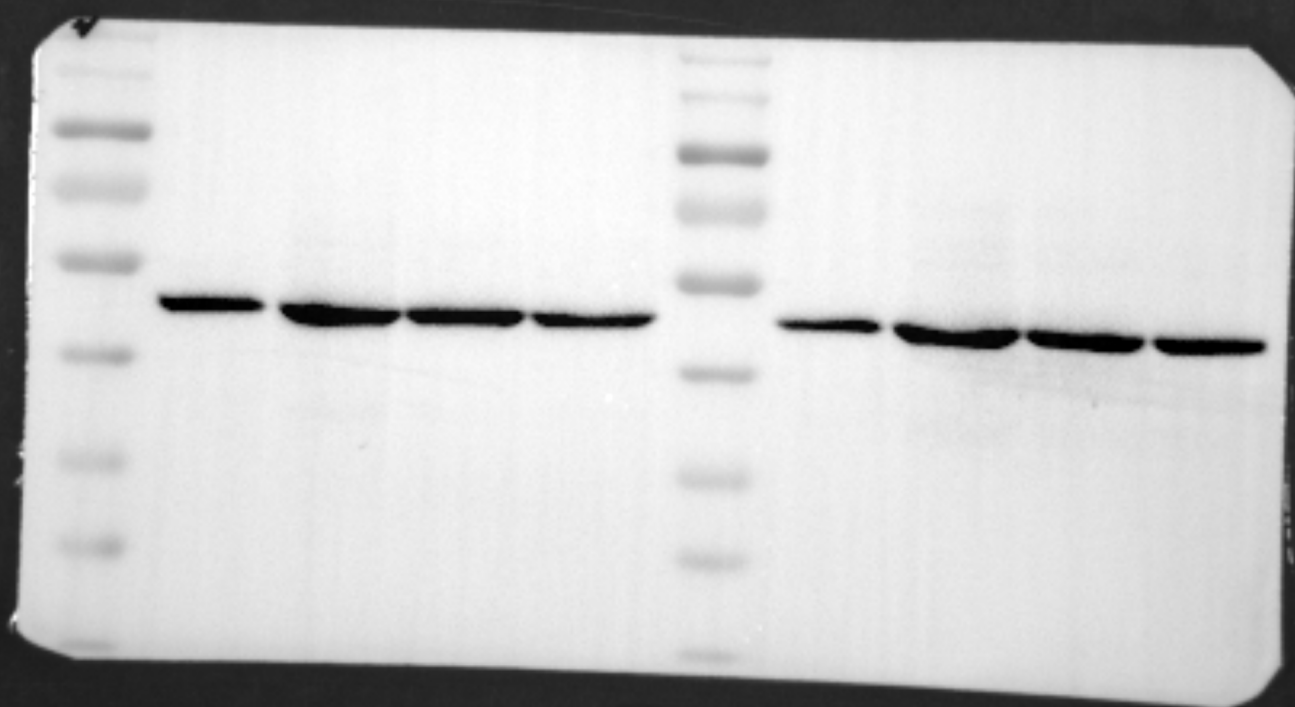

Supplement: Supplementary file 1 — Supplementary Information 1. [file 41598_2022_19614_MOESM1_ESM.pdf]

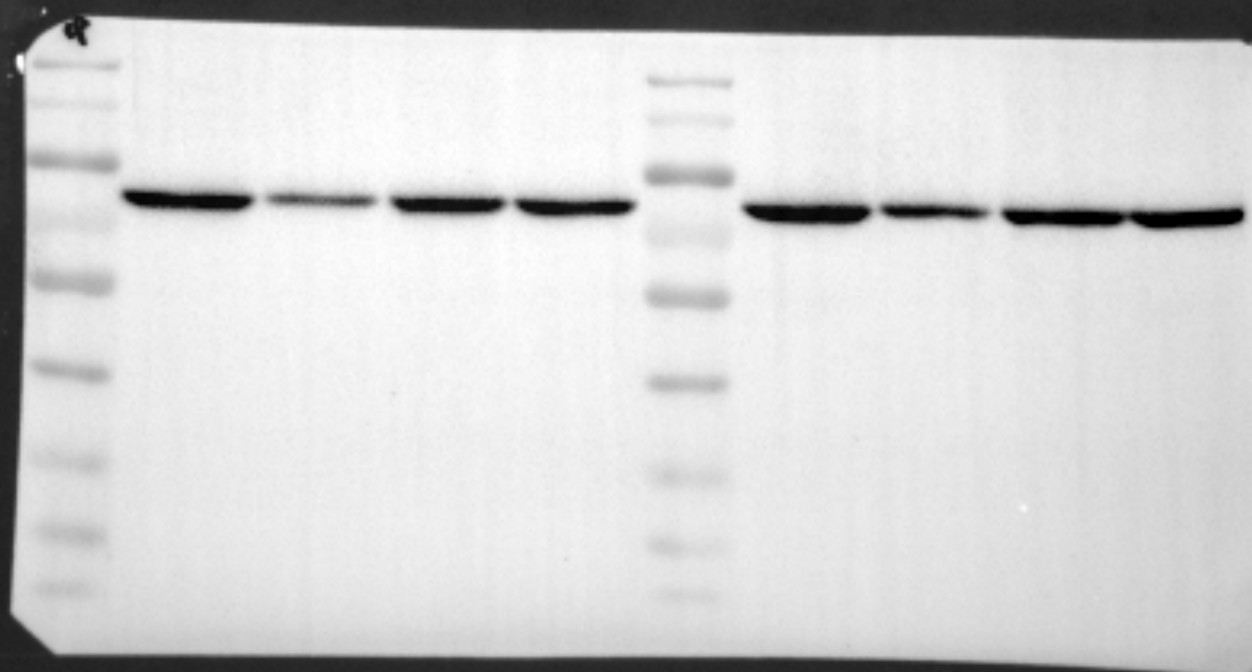

Supplement: Supplementary file 2 — Supplementary Information 2. [file 41598_2022_19614_MOESM2_ESM.pdf]

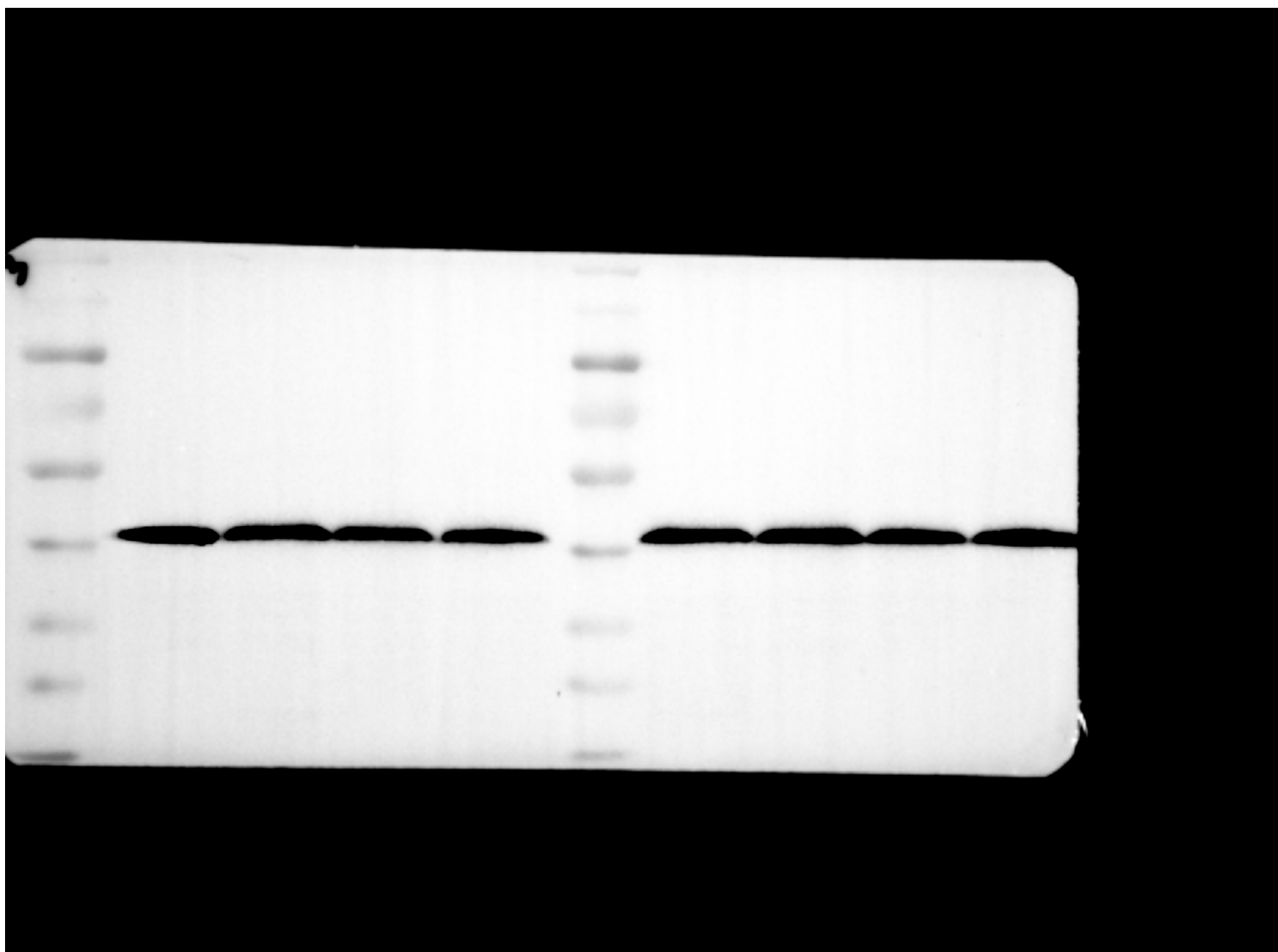

Supplement: Supplementary file 3 — Supplementary Information 3. [file 41598_2022_19614_MOESM3_ESM.pdf]

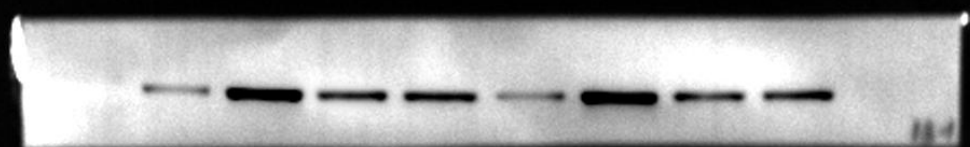

Supplement: Supplementary file 4 — Supplementary Information 4. [file 41598_2022_19614_MOESM4_ESM.pdf]

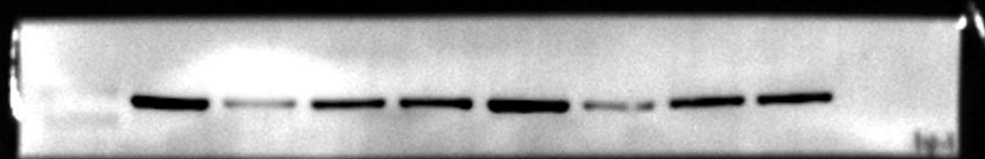

Supplement: Supplementary file 5 — Supplementary Information 5. [file 41598_2022_19614_MOESM5_ESM.pdf]

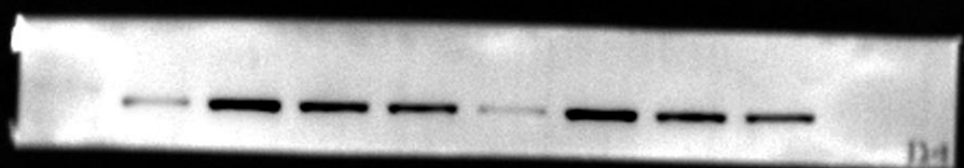

Supplement: Supplementary file 6 — Supplementary Information 6. [file 41598_2022_19614_MOESM6_ESM.pdf]

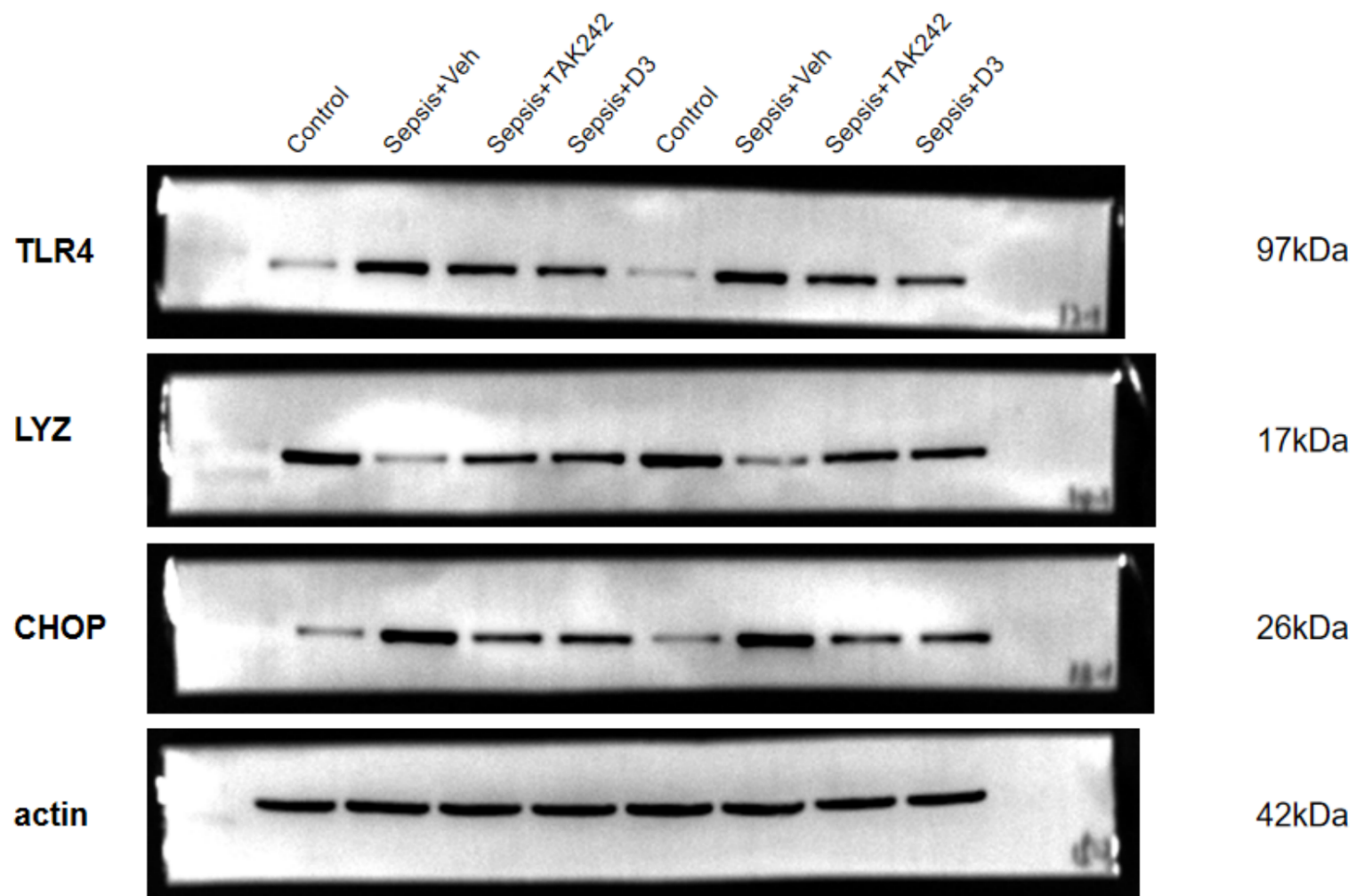

Supplement: Supplementary file 7 — Supplementary Information 7. [file 41598_2022_19614_MOESM7_ESM.pdf]

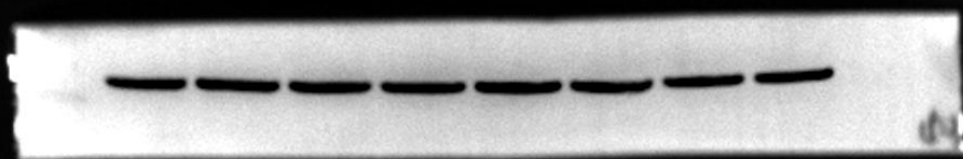

Supplement: Supplementary file 8 — Supplementary Information 8. [file 41598_2022_19614_MOESM8_ESM.pdf]
